# Sodium (1*R*)-d-glucit-1-yl­sulfonate monohydrate

**DOI:** 10.1107/S1600536812007210

**Published:** 2012-03-07

**Authors:** Alan H. Haines, David L. Hughes

**Affiliations:** a School of Chemistry, University of East Anglia, Norwich NR4 7TJ, England

## Abstract

The title salt, Na^+^·C_6_H_13_O_9_S^−^·H_2_O, crystallizes with three independent cations, molecular anions and solvent water molecules in the asymmetric unit. This crystalline monohydrate addition product, formed by reaction of d-glucose and sodium hydrogen sulfite in water, forms a three-dimensional network through complex cation coordination and extensive inter­molecular hydrogen bonding. Each of the independent mol­ecules has an open-chain structure with the carbon chains adopting a sickle-like conformation, similar to that found in the potassium salt [Cole *et al.* (2001 ▶). *Carbohydr. Res.* **335**, 1–10], but there are significant differences in the patterns of complexation.

## Related literature

For the first syntheses of the title compound, see: Braverman (1953 ▶); Ingles (1959 ▶). For evidence of the acyclic nature of such compounds, see: Ingles (1959 ▶, 1969 ▶). For the synthesis and crystallographic properties of the corresponding potassium salts of d-glucose and d-mannose, see: Cole *et al.* (2001 ▶). For an additional discussion on the potassium salt, see: Haines & Hughes (2010 ▶). For the crystallographic study of potassium (1*S*)-d-galactit-1-yl­sulfonate, see: Haines & Hughes (2010 ▶).
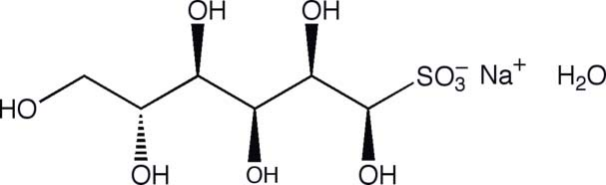


## Experimental

### 

#### Crystal data

Na^+^·C_6_H_13_O_9_S^−^·H_2_O
*M* *_r_* = 302.23
Orthorhombic, 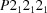

*a* = 8.81958 (9) Å
*b* = 16.8420 (2) Å
*c* = 22.7304 (3) Å
*V* = 3376.37 (7) Å^3^
*Z* = 12
Mo *K*α radiation
μ = 0.37 mm^−1^
*T* = 140 K
0.30 × 0.19 × 0.13 mm

#### Data collection

Oxford Diffraction Xcalibur 3/Sapphire3 CCD diffractometer
Absorption correction: multi-scan (*CrysAlis RED*; Oxford Diffraction, 2008 ▶) *T*_min_ = 0.908, *T*_max_ = 1.000
68733 measured reflections
9835 independent reflections
8207 reflections with *I* > 2σ(*I*)
*R*_int_ = 0.044

#### Refinement

*R*[*F* ^2^ > 2σ(*F* ^2^)] = 0.028
*wR*(*F* ^2^) = 0.052
*S* = 0.93
9835 reflections
583 parameters
H atoms treated by a mixture of independent and constrained refinement
Δρ_max_ = 0.53 e Å^−3^
Δρ_min_ = −0.46 e Å^−3^
Absolute structure: Flack (1983 ▶), 4356 Friedel pairs
Flack parameter: 0.02 (3)

### 

Data collection: *CrysAlis PRO* (Oxford Diffraction, 2010 ▶); cell refinement: *CrysAlis PRO*; data reduction: *CrysAlis PRO*; program(s) used to solve structure: *SHELXS97* (Sheldrick, 2008 ▶); program(s) used to refine structure: *SHELXL97* (Sheldrick, 2008 ▶); molecular graphics: *ORTEPII* (Johnson, 1976 ▶) and *ORTEP-3* (Farrugia, 1997 ▶); software used to prepare material for publication: *SHELXL97* and *WinGX* (Farrugia, 1999 ▶).

## Supplementary Material

Crystal structure: contains datablock(s) I, global. DOI: 10.1107/S1600536812007210/su2377sup1.cif

Supplementary material file. DOI: 10.1107/S1600536812007210/su2377Isup2.cdx

Structure factors: contains datablock(s) I. DOI: 10.1107/S1600536812007210/su2377Isup3.hkl

Supplementary material file. DOI: 10.1107/S1600536812007210/su2377Isup4.cml

Additional supplementary materials:  crystallographic information; 3D view; checkCIF report

## Figures and Tables

**Table 1:** Hydrogen-bond geometry (Å, °)

*D*—H⋯*A*	*D*—H	H⋯*A*	*D*⋯*A*	*D*—H⋯*A*
O1—H1O⋯O13	0.81 (2)	2.21 (2)	2.8719 (17)	139 (2)
O1—H1O⋯O12	0.81 (2)	2.23 (2)	2.9262 (16)	144 (2)
O2—H2O⋯O33^i^	0.770 (17)	2.243 (19)	2.927 (2)	148.5 (17)
O3—H3O⋯O15^ii^	0.813 (19)	1.989 (19)	2.7862 (15)	166.9 (19)
O4—H4O⋯O24^iii^	0.84 (2)	2.24 (2)	3.0150 (16)	153.0 (19)
O5—H5O⋯O16^ii^	0.79 (2)	1.85 (2)	2.6172 (16)	164 (2)
O6—H6O⋯O7^iv^	0.76 (2)	2.06 (2)	2.8035 (17)	166 (2)
O11—H11O⋯O22^v^	0.80 (2)	2.39 (2)	3.0578 (16)	141 (2)
O11—H11O⋯O23^v^	0.80 (2)	2.12 (2)	2.7906 (17)	142 (2)
O12—H12O⋯O9	0.81 (2)	2.05 (2)	2.8372 (15)	165.7 (19)
O13—H13O⋯O25^vi^	0.768 (18)	2.082 (19)	2.8027 (16)	156.5 (19)
O14—H14O⋯O4^iii^	0.77 (2)	2.18 (2)	2.9173 (16)	162 (2)
O15—H15O⋯O25^vi^	0.805 (19)	1.939 (19)	2.7296 (16)	167 (2)
O16—H16O⋯O32^vi^	0.74 (2)	2.00 (2)	2.7346 (18)	173 (2)
O21—H21O⋯O2	0.84 (2)	2.13 (2)	2.9010 (16)	153.9 (19)
O21—H21O⋯O3	0.84 (2)	2.28 (2)	2.8810 (17)	129.4 (18)
O22—H22O⋯O19^vii^	0.765 (18)	1.995 (18)	2.7576 (15)	175 (2)
O23—H23O⋯O5^ii^	0.83 (2)	2.01 (2)	2.8219 (15)	168 (2)
O24—H24O⋯O14^viii^	0.76 (2)	2.34 (2)	3.0445 (16)	156.3 (19)
O25—H25O⋯O5^ii^	0.777 (19)	1.898 (19)	2.6709 (15)	173 (2)
O26—H26O⋯O31^ii^	0.76 (2)	2.14 (2)	2.8463 (18)	155 (2)
O31—H31*A*⋯O18^ix^	0.700 (19)	2.08 (2)	2.7729 (17)	170 (2)
O31—H31*B*⋯O6^vi^	0.83 (2)	2.07 (2)	2.8493 (18)	156 (2)
O32—H32*A*⋯O26^vi^	0.89 (2)	1.89 (2)	2.7501 (17)	163 (2)
O32—H32*B*⋯O8^ix^	0.741 (18)	2.019 (18)	2.7471 (16)	167.1 (17)
O33—H33*A*⋯O27^x^	0.833 (19)	2.16 (2)	2.9093 (18)	149.9 (19)
O33—H33*B*⋯O28^ix^	0.80 (3)	2.02 (3)	2.7371 (17)	150 (2)

Symmetry codes: (i) 
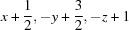
; (ii) 
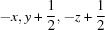
; (iii) 
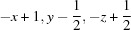
; (iv) 
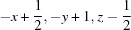
; (v) 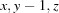; (vi) 
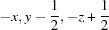
; (vii) 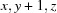; (viii) 
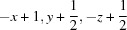
; (ix) 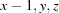; (x) 
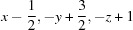
.

**Fig. 1.**
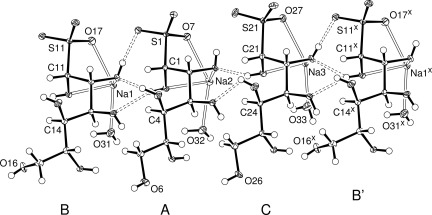
A view of the three, unique, linked complexes A, B, C (plus an adjacent group, B1) of the title compound, indicating the atom numbering scheme. The groups of three complexes are repeated by translation parallel to the b axis. Thermal ellipsoids are drawn at the 50% probability level. Symmetry code: (x) x, y+1, z.

**Fig. 2.**
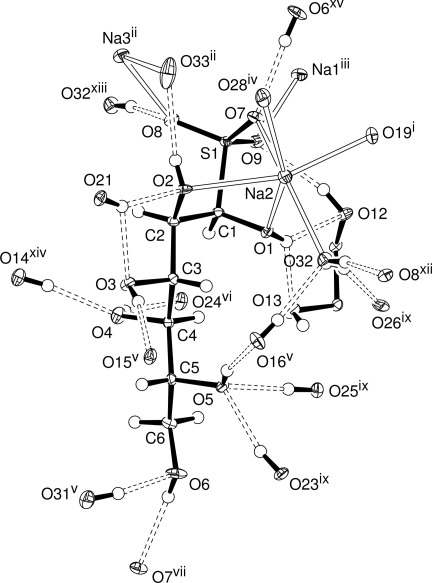
One of the three sugar units, A, with all its sodium ion contacts and hydrogen bond interactions (dashed double lines). The atom numbering scheme is shown; the atoms of the other molecules are numbered correspondingly, with C, O and S atom numbers n+10 and n+20. The water molecules are labelled O31, O32 and O33. Symmetry codes: (i) x-1/2, -y+1/2, -z+1; (ii) x+1/2, -y+3/2, -z+1; (iii) x+1/2, -y+1/2, -z+1; (iv) x-1/2, -y+3/2, -z+1; (v) -x, y+1/2, -z+1/2; (vi) -x+1, y-1/2, -z+1/2; (vii) -x+1/2, -y+1, z-1/2; (ix) -x, y-1/2, -z+1/2; (xii) x-1, y, z; (xiii) x+1, y, z; (xiv) -x+1, y+1/2, -z+1/2; (xv) -x+1/2, -y+1, z+1/2.

**Fig. 3.**
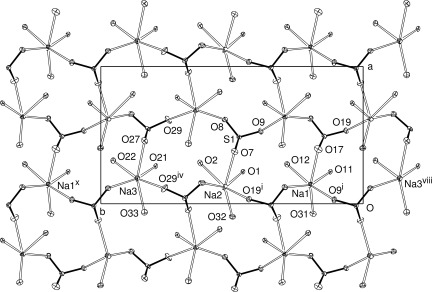
View down the c axis, showing the sodium ions, lying in a sheet parallel to (001), with all their coordinated atoms linked by bridging sulfonate groups. Symmetry codes: (i) x-1/2, -y+1/2, -z+1; (iv) x-1/2, -y+3/2, -z+1; (viii) x, y-1, z; (x) x, y+1, z.

## supplementary crystallographic information

### Comment

The addition of bisulfite anion to carbonyl compounds has found use in the purification of aldehydes and some ketones. That aldoses, despite existing preponderantly in the hemi-acetal form, also form such adducts, has been known for over a century and evidence was provided by Ingles (1959, 1969) for the acyclic nature of such compounds but the open-chain form of the crystalline sulfonic salts derived from D-glucose and D-mannose with potassium bisulfite was only proved conclusively more recently by X-ray crystallographic studies (Cole *et al.*, 2001). A study on the D-galactose compound (Haines & Hughes, 2010) also proved its acyclic nature.

Storage of a concentrated aqueous solution of D-glucose and equimolar sodium bisulfite (generated in the aqueous solution from sodium metabisulfite) at 277 K for several months, gave crystals of sodium (1*R*)-D-glucit-1-ylsulfonate monohydrate, 1, with properties (mp and [α]_D_) in agreement with those reported (Braverman, 1953; Ingles, 1959). HRESIMS (negative ion mode) indicated a peak at 261.0284 (calcd for [C_6_H_13_O_9_S]^-^: m/*z* 261.0286).

The title adduct (Fig. 1) crystallizes with three independent molecules per asymmetric unit; in contrast, the potassium adduct, also a monohydrate, has only one (Cole *et al.*, 2001). Each of the three molecules adopts a sickle-like conformation with *gauche* conformations in the region C1—C2—C3—C4. Other torsion angles in the chains (which include the sulfur atom), all have values close to 180°, *i.e.* with *anti* conformations. Molecule B differs from A and C in having atom O16 approximately *anti* to H15; in A and C, atoms O6 and H5 adopt a *gauche* relationship about the C5–C6 bond.

In the crystal, the groups of three molecules, A, B, and C, are repeated by translation parallel to the *b* axis (Fig. 1).

The potassium compound also has the *R* configuration at C1 (Cole *et al.*, 2001) but coordination of the sodium cation is distinctly different from that around the potassium ion. The sodium ions are each hexa-coordinated with oxygen atoms in the title compound [see Fig. 2 for the coordination pattern of Na2], with three different carbohydrate ligands providing five O atoms and a water molecule the sixth. For each sodium ion, one carbohydrate residue provides three of these O atoms, O1, O2 and a sulfonate oxygen O7; the other two oxygen atoms are provided by sulfonate O atoms from the two other residues. In contrast, the potassium compound has the cation coordinated to seven O atoms which are provided by four different carbohydrate molecules and a water molecule (Cole *et al.*, 2001; Haines & Hughes, 2010).

Extensive intermolecular hydrogen bonding involves all three of the distinct anions, and this is indicated for one anion in Fig. 2. Every hydroxyl group is involved as a donor group in a hydrogen bond, and all except those at C1 (which are coordinated to sodium ions) are acceptors. The hydrogen atom H1O, of the hydroxy group at C1 of each anion (see Table 1), is involved in a bifurcated hydrogen bond to oxygen atoms of the hydroxy groups at C2 and C3 in an adjacent molecule (Figs. 1 and 2). The hydrogen bonds of the OH groups at C3 and C4 in each molecule are directed to oxygen atoms O5 and O4 in adjacent molecules. From molecules B and C, the hydrogen bonds involving atoms O3 and O5 are both accepted by O5 of an adjacent molecule, whereas the corresponding bonds from O3 and O5 of molecule A are accepted by O5 and O6, respectively, of the adjacent molecule. The remaining OH groups are linked less regularly, but all are involved in hydrogen bonds to main-chain OH groups, sulfonate O atoms or water molecules.

The three water molecules all have an approximately tetrahedral bonding pattern. Each water O atom is coordinated with a sodium ion and bonded to two hydrogen atoms, one of which forms a hydrogen bond to an O8 sulfonate atom, the other to either an O6 atom or an O7 sulfonate atom; the fourth site is the acceptor end of a hydrogen bond. This is shown for atom O32 in Fig. 2.

A simplified view along the crystallographic *c* axis (Fig. 3) shows the remarkable way in which a network of sulfonate residues is linearly linked (parallel to the *b* axis) through two of their oxygen atoms, O8 and O9, by rows of sodium atoms; cross-links, parallel to the *a* axis, between these chains are made through the third of the sulfonate oxygen atoms, O7. The complex coordination and hydrogen bonding leads to a complex, extensive, three-dimensional network.

### Experimental

The title compound was prepared by a modification of previous procedures (Braverman, 1953; Ingles, 1959). Crystals of the title compound were obtained by storage of a solution of D-glucose (1.8 g) and sodium metabisulfite (0.95 g) in water (2 ml) at ~277 K for several weeks. The crystals, washed with MeOH:H_2_O (4:1) and dried over P_2_O_5_, had a non-sharp m.p. of 366-369 K [lit. 365-366 K (Braverman, 1953) and 372 K (Ingles, 1959)]; [α]_D_^25^ -5.2 (*c* 2.22, 9:1 H_2_O:HOAc), (lit. [α]_D_^25^ -3.9 (*c* 3.8, 9:1 H_2_O:HOAc); Ingles 1959). HRESIMS (negative ion mode) indicated a peak at 261.0284 (calcd for [C_6_H_13_O_9_S]^-^: m/*z* 261.0286). Further spectroscopic data are given in the archived CIF.

### Refinement

The hydroxyl H atoms were located in difference Fourier maps and were freely refined. The C-bound H atoms were included in calculated positions and treated as riding atoms: C-H = 0.98 and 0.97 Å for CH and CH_2_ H atoms, respectively, with *U*_iso_(H)= 1.2*U*_eq_(C).

### Crystal data

**Table:**

Na^+^·C_6_H_13_O_9_S^−^·H_2_O	*F*(000) = 1896
*M**_r_* = 302.23	*D*_x_ = 1.784 Mg m^−^^3^
Orthorhombic, *P*2_1_2_1_2_1_	Mo *K*α radiation, λ = 0.71073 Å
Hall symbol: P 2ac 2ab	Cell parameters from 27478 reflections
*a* = 8.81958 (9) Å	θ = 3.5–32.6°
*b* = 16.8420 (2) Å	µ = 0.37 mm^−^^1^
*c* = 22.7304 (3) Å	*T* = 140 K
*V* = 3376.37 (7) Å^3^	Prism, colourless
*Z* = 12	0.30 × 0.19 × 0.13 mm

### Data collection

**Table:**

Oxford Diffraction Xcalibur 3/Sapphire3 CCD diffractometer	9835 independent reflections
Radiation source: Enhance (Mo) X-ray Source	8207 reflections with *I* > 2σ(*I*)
Graphite monochromator	*R*_int_ = 0.044
Detector resolution: 16.0050 pixels mm^-1^	θ_max_ = 30°, θ_min_ = 3.5°
Thin slice φ and ω scans	*h* = −12→12
Absorption correction: multi-scan (*CrysAlis RED*; Oxford Diffraction, 2008)	*k* = −23→23
*T*_min_ = 0.908, *T*_max_ = 1.000	*l* = −31→31
68733 measured reflections	

### Refinement

**Table:**

Refinement on *F*^2^	Secondary atom site location: difference Fourier map
Least-squares matrix: full	Hydrogen site location: inferred from neighbouring sites
*R*[*F*^2^ > 2σ(*F*^2^)] = 0.028	H atoms treated by a mixture of independent and constrained refinement
*wR*(*F*^2^) = 0.052	*w* = 1/[σ^2^(*F*_o_^2^) + (0.0248*P*)^2^] where *P* = (*F*_o_^2^ + 2*F*_c_^2^)/3
*S* = 0.93	(Δ/σ)_max_ = 0.001
9835 reflections	Δρ_max_ = 0.53 e Å^−^^3^
583 parameters	Δρ_min_ = −0.46 e Å^−^^3^
0 restraints	Absolute structure: Flack (1983), 4356 Friedel pairs
Primary atom site location: structure-invariant direct methods	Flack parameter: 0.02 (3)

### Special details

**Table:**

Experimental. Absorption correction: CrysAlis RED (Oxford Diffraction, 2008). Empirical absorption correction using spherical harmonics, implemented in SCALE3 ABSPACK scaling algorithm.Spectroscopic data for the title compound:^1^H NMR (D_2_O, 300 MHz, measured 5 min after dissolution, reference *Me*_3_COH at δ_H_ 1.24): δ 5.23 (d, *J* 2.8 Hz, OCHO of α-pyranose), 4.64 (d, *J* 8.2 Hz, OCHO of β-pyranose), 4.53 (br s, CH[SO_3_^-^]), δ 4.48 (d, *J* 6.9 Hz, CH[SO_3_^-^]), 4.25–3.30 (complex), 3.23 (dd, *J*_3,4_ = *J*_4,5_ = 8.3 Hz, H-4); after 24 h the spectrum was of a mixture of the α- and β-pyranoses. ^13^C NMR (D_2_O, 75 MHz, scan time 17 min, referenced to *Me*_3_COH at δ_C_ 30.29): δ 96.62 (β-pyranose C1), 92.82 (α-pyranose C1), 84.10 (CH[SO_3_^-^]), 82.87 (CH[SO_3_^-^]), 76.63 - 61.27 (16 signals).HRESIMS (negative ion mode): calcd for [C_6_H_13_O_9_S]^-^: *m/z* 261.0286; found 261.0284; predominant peaks were also observed at *m/z* 179.0567 ([C_6_H_11_O_6_]^-^), 243.0181 ([C_6_H_13_SO_9_ - H_2_O]^-^) and 359.1196 ([C_12_H_23_O_12_]^-^). The latter corresponds to the ion of the product formed by reaction between the sulfonate and D-glucose with displacement of sodium bisulfite; some decomposition of the sulfonate to afford D-glucose undoubtedly occurs in aqueous solution.
Geometry. All s.u.'s (except the s.u. in the dihedral angle between two l.s. planes) are estimated using the full covariance matrix. The cell s.u.'s are taken into account individually in the estimation of s.u.'s in distances, angles and torsion angles; correlations between s.u.'s in cell parameters are only used when they are defined by crystal symmetry. An approximate (isotropic) treatment of cell s.u.'s is used for estimating s.u.'s involving l.s. planes.
Refinement. Refinement of *F*^2^ against ALL reflections. The weighted *R*-factor *wR* and goodness of fit *S* are based on *F*^2^, conventional *R*-factors *R* are based on *F*, with *F* set to zero for negative *F*^2^. The threshold expression of *F*^2^ > 2σ(*F*^2^) is used only for calculating *R*-factors(gt) *etc*. and is not relevant to the choice of reflections for refinement. *R*-factors based on *F*^2^ are statistically about twice as large as those based on *F*, and *R*- factors based on ALL data will be even larger.

### Fractional atomic coordinates and isotropic or equivalent isotropic displacement parameters (Å^2^)

**Table:**

	*x*	*y*	*z*	*U*_iso_*/*U*_eq_	
Na1	0.13614 (6)	0.20117 (4)	0.48122 (3)	0.01344 (14)	
C1	0.37924 (17)	0.49133 (9)	0.38737 (7)	0.0089 (3)	
H1	0.4307	0.4649	0.3545	0.011*	
O1	0.23320 (11)	0.45878 (7)	0.39417 (5)	0.0107 (2)	
C2	0.36436 (16)	0.57970 (9)	0.37396 (7)	0.0091 (3)	
H2	0.4643	0.6022	0.3651	0.011*	
O2	0.29707 (13)	0.62124 (6)	0.42243 (5)	0.0121 (2)	
C3	0.25670 (16)	0.59384 (9)	0.32241 (7)	0.0090 (3)	
H3	0.154	0.579	0.3347	0.011*	
O3	0.25886 (13)	0.67734 (7)	0.31079 (5)	0.0125 (2)	
C4	0.29518 (15)	0.54829 (9)	0.26626 (7)	0.0089 (3)	
H4	0.2991	0.4914	0.2752	0.011*	
O4	0.44056 (12)	0.57338 (7)	0.24505 (5)	0.0145 (2)	
C5	0.17485 (16)	0.56284 (9)	0.21839 (7)	0.0086 (3)	
H5	0.1701	0.6197	0.2093	0.01*	
O5	0.02947 (11)	0.53644 (7)	0.23940 (5)	0.0112 (2)	
C6	0.20478 (17)	0.51686 (9)	0.16275 (7)	0.0130 (3)	
H6A	0.2987	0.5344	0.1448	0.016*	
H6B	0.2131	0.4606	0.1713	0.016*	
O6	0.08016 (13)	0.53109 (8)	0.12399 (5)	0.0186 (3)	
S1	0.48430 (4)	0.47327 (2)	0.454827 (17)	0.00845 (7)	
O7	0.38035 (12)	0.48990 (6)	0.50260 (5)	0.0141 (2)	
O8	0.61356 (11)	0.52732 (6)	0.45317 (5)	0.0129 (2)	
O9	0.52894 (11)	0.38984 (6)	0.45184 (5)	0.0134 (2)	
O31	−0.07200 (14)	0.19093 (8)	0.41695 (6)	0.0182 (3)	
Na2	0.12042 (7)	0.52742 (4)	0.47754 (3)	0.01419 (14)	
C11	0.37470 (16)	0.16002 (8)	0.38419 (7)	0.0085 (3)	
H11	0.4221	0.1316	0.3514	0.01*	
O11	0.23253 (12)	0.12558 (7)	0.39667 (5)	0.0117 (2)	
C12	0.35304 (16)	0.24679 (9)	0.36746 (7)	0.0099 (3)	
H12	0.4515	0.2701	0.3575	0.012*	
O12	0.28739 (12)	0.28985 (7)	0.41568 (5)	0.0130 (2)	
C13	0.24380 (16)	0.25754 (9)	0.31569 (7)	0.0089 (3)	
H13	0.1417	0.2418	0.3281	0.011*	
O13	0.24430 (14)	0.34098 (6)	0.30291 (6)	0.0146 (3)	
C14	0.28632 (15)	0.21128 (9)	0.26046 (7)	0.0097 (3)	
H14	0.2834	0.1542	0.2689	0.012*	
O14	0.43666 (12)	0.23329 (7)	0.24221 (5)	0.0150 (2)	
C15	0.17976 (17)	0.22993 (9)	0.20861 (7)	0.0107 (3)	
H15	0.2049	0.2829	0.1937	0.013*	
O15	0.02371 (12)	0.23051 (7)	0.22733 (5)	0.0141 (2)	
C16	0.19286 (17)	0.17162 (9)	0.15842 (7)	0.0132 (3)	
H16A	0.1321	0.1896	0.1254	0.016*	
H16B	0.2976	0.1682	0.1456	0.016*	
O16	0.14154 (13)	0.09536 (7)	0.17724 (6)	0.0153 (3)	
S2	0.49099 (4)	0.14863 (2)	0.449704 (18)	0.00980 (8)	
O17	0.39821 (12)	0.17128 (7)	0.49950 (5)	0.0190 (3)	
O18	0.62086 (11)	0.20125 (6)	0.44188 (5)	0.0163 (2)	
O19	0.53301 (11)	0.06453 (6)	0.45018 (5)	0.0135 (2)	
O32	−0.09392 (14)	0.49853 (7)	0.41754 (6)	0.0157 (3)	
Na3	0.18414 (7)	0.86152 (4)	0.48930 (3)	0.01404 (14)	
C21	0.41018 (15)	0.82810 (9)	0.38808 (7)	0.0103 (3)	
H21	0.4517	0.8011	0.3535	0.012*	
O21	0.27321 (12)	0.79143 (7)	0.40507 (5)	0.0130 (2)	
C22	0.37895 (16)	0.91475 (9)	0.37330 (7)	0.0097 (3)	
H22	0.4741	0.9411	0.3626	0.012*	
O22	0.31453 (13)	0.95299 (7)	0.42371 (5)	0.0125 (2)	
C23	0.26528 (16)	0.92370 (9)	0.32343 (7)	0.0100 (3)	
H23	0.1658	0.9056	0.3372	0.012*	
O23	0.25714 (14)	1.00715 (7)	0.31134 (5)	0.0136 (2)	
C24	0.30638 (16)	0.87809 (9)	0.26775 (7)	0.0094 (3)	
H24	0.3106	0.8212	0.2767	0.011*	
O24	0.45210 (12)	0.90377 (7)	0.24678 (5)	0.0156 (2)	
C25	0.19041 (16)	0.89229 (9)	0.21883 (7)	0.0093 (3)	
H25	0.2031	0.9461	0.2032	0.011*	
O25	0.03936 (11)	0.88392 (7)	0.24267 (5)	0.0117 (2)	
C26	0.20396 (17)	0.83301 (9)	0.16940 (7)	0.0140 (3)	
H26A	0.301	0.8389	0.1498	0.017*	
H26B	0.1968	0.7794	0.1847	0.017*	
O26	0.08309 (13)	0.84798 (7)	0.12910 (5)	0.0176 (3)	
S3	0.54392 (4)	0.82111 (2)	0.448640 (17)	0.01092 (8)	
O27	0.45870 (12)	0.83333 (6)	0.50307 (5)	0.0156 (2)	
O28	0.65285 (11)	0.88547 (7)	0.43851 (5)	0.0175 (3)	
O29	0.61220 (12)	0.74280 (6)	0.44472 (5)	0.0186 (3)	
O33	−0.06144 (15)	0.83394 (8)	0.46595 (7)	0.0323 (4)	
H1O	0.234 (2)	0.4124 (14)	0.3846 (10)	0.041 (7)*	
H2O	0.3615 (19)	0.6249 (10)	0.4451 (8)	0.015 (5)*	
H3O	0.172 (2)	0.6912 (11)	0.3051 (8)	0.023 (5)*	
H4O	0.497 (2)	0.5334 (12)	0.2423 (9)	0.031 (6)*	
H5O	−0.011 (3)	0.5613 (14)	0.2642 (10)	0.050 (8)*	
H6O	0.099 (2)	0.5188 (12)	0.0927 (9)	0.028 (6)*	
H31A	−0.148 (2)	0.1898 (12)	0.4262 (9)	0.020 (6)*	
H31B	−0.048 (3)	0.1479 (14)	0.4021 (11)	0.056 (8)*	
H11O	0.240 (2)	0.0802 (14)	0.3868 (10)	0.045 (7)*	
H12O	0.357 (2)	0.3131 (12)	0.4310 (9)	0.028 (6)*	
H13O	0.161 (2)	0.3537 (11)	0.3003 (8)	0.018 (5)*	
H14O	0.484 (2)	0.1952 (13)	0.2408 (9)	0.030 (6)*	
H15O	0.002 (2)	0.2767 (12)	0.2307 (8)	0.023 (6)*	
H16O	0.136 (2)	0.0694 (12)	0.1511 (10)	0.031 (7)*	
H32A	−0.078 (2)	0.4475 (12)	0.4088 (9)	0.036 (6)*	
H32B	−0.171 (2)	0.5006 (9)	0.4305 (8)	0.005 (5)*	
H21O	0.273 (2)	0.7427 (13)	0.3984 (9)	0.031 (6)*	
H22O	0.375 (2)	0.9831 (11)	0.4333 (8)	0.020 (6)*	
H23O	0.170 (2)	1.0184 (12)	0.3015 (10)	0.037 (7)*	
H24O	0.503 (2)	0.8676 (12)	0.2473 (9)	0.024 (6)*	
H25O	0.013 (2)	0.9270 (11)	0.2492 (9)	0.017 (5)*	
H26O	0.078 (3)	0.8135 (13)	0.1074 (10)	0.050 (8)*	
H33A	−0.073 (2)	0.7851 (12)	0.4627 (9)	0.028 (6)*	
H33B	−0.130 (3)	0.8490 (14)	0.4458 (12)	0.060 (9)*	

### Atomic displacement parameters (Å^2^)

**Table:**

	*U*^11^	*U*^22^	*U*^33^	*U*^12^	*U*^13^	*U*^23^
Na1	0.0146 (3)	0.0141 (3)	0.0116 (3)	−0.0019 (2)	0.0030 (2)	−0.0002 (3)
C1	0.0093 (7)	0.0095 (7)	0.0079 (8)	0.0004 (5)	−0.0027 (6)	0.0001 (6)
O1	0.0106 (5)	0.0085 (5)	0.0131 (6)	−0.0017 (4)	−0.0014 (4)	−0.0009 (5)
C2	0.0109 (7)	0.0089 (7)	0.0075 (8)	0.0001 (5)	−0.0008 (6)	−0.0011 (6)
O2	0.0163 (6)	0.0113 (6)	0.0086 (6)	0.0035 (4)	−0.0051 (5)	−0.0047 (4)
C3	0.0092 (6)	0.0076 (7)	0.0101 (8)	0.0004 (5)	−0.0008 (6)	0.0003 (6)
O3	0.0143 (5)	0.0065 (5)	0.0167 (6)	0.0021 (5)	−0.0058 (5)	0.0003 (5)
C4	0.0088 (6)	0.0075 (7)	0.0103 (8)	−0.0001 (5)	0.0007 (5)	0.0006 (6)
O4	0.0082 (5)	0.0168 (6)	0.0184 (6)	0.0011 (5)	0.0028 (5)	−0.0010 (5)
C5	0.0082 (6)	0.0089 (7)	0.0085 (8)	−0.0008 (6)	0.0011 (6)	0.0009 (6)
O5	0.0089 (5)	0.0150 (5)	0.0096 (6)	−0.0028 (4)	0.0019 (4)	−0.0036 (5)
C6	0.0146 (8)	0.0153 (8)	0.0092 (8)	0.0012 (6)	−0.0001 (6)	0.0002 (6)
O6	0.0208 (6)	0.0286 (7)	0.0064 (6)	0.0041 (5)	−0.0028 (5)	−0.0042 (6)
S1	0.00888 (16)	0.00855 (16)	0.00793 (18)	0.00076 (13)	−0.00175 (15)	0.00017 (15)
O7	0.0145 (5)	0.0194 (6)	0.0085 (6)	0.0036 (4)	0.0002 (5)	0.0009 (5)
O8	0.0108 (5)	0.0141 (5)	0.0136 (6)	−0.0004 (4)	−0.0026 (5)	−0.0014 (5)
O9	0.0169 (5)	0.0096 (5)	0.0136 (6)	0.0016 (4)	−0.0055 (5)	0.0008 (5)
O31	0.0137 (6)	0.0186 (7)	0.0224 (7)	0.0022 (5)	0.0010 (5)	−0.0006 (5)
Na2	0.0138 (3)	0.0165 (3)	0.0122 (3)	−0.0020 (3)	0.0009 (3)	0.0005 (3)
C11	0.0094 (6)	0.0094 (8)	0.0068 (7)	0.0010 (5)	−0.0014 (6)	−0.0004 (6)
O11	0.0111 (5)	0.0088 (6)	0.0151 (6)	−0.0023 (4)	−0.0003 (4)	−0.0020 (5)
C12	0.0089 (7)	0.0091 (7)	0.0116 (8)	−0.0006 (5)	−0.0013 (6)	−0.0020 (6)
O12	0.0137 (6)	0.0117 (6)	0.0134 (6)	0.0010 (4)	−0.0023 (4)	−0.0062 (5)
C13	0.0086 (6)	0.0075 (7)	0.0107 (8)	0.0002 (5)	−0.0008 (6)	−0.0003 (6)
O13	0.0139 (6)	0.0077 (6)	0.0223 (7)	0.0021 (5)	−0.0075 (5)	0.0007 (5)
C14	0.0082 (6)	0.0076 (7)	0.0133 (8)	−0.0014 (5)	0.0015 (6)	0.0005 (6)
O14	0.0081 (5)	0.0169 (6)	0.0199 (7)	0.0008 (5)	0.0025 (5)	0.0009 (5)
C15	0.0102 (7)	0.0109 (7)	0.0110 (8)	−0.0010 (6)	0.0003 (6)	0.0025 (6)
O15	0.0082 (5)	0.0133 (6)	0.0207 (6)	0.0011 (4)	−0.0031 (5)	−0.0048 (5)
C16	0.0156 (7)	0.0149 (8)	0.0091 (8)	−0.0027 (6)	0.0008 (6)	0.0029 (7)
O16	0.0229 (6)	0.0110 (6)	0.0120 (6)	−0.0034 (5)	0.0055 (5)	−0.0026 (5)
S2	0.01007 (16)	0.00945 (16)	0.00987 (19)	0.00082 (13)	−0.00216 (16)	−0.00123 (16)
O17	0.0206 (6)	0.0279 (7)	0.0086 (6)	0.0064 (5)	−0.0010 (5)	−0.0031 (5)
O18	0.0131 (5)	0.0137 (6)	0.0221 (7)	−0.0024 (4)	−0.0055 (5)	0.0000 (5)
O19	0.0141 (5)	0.0113 (5)	0.0149 (6)	0.0011 (4)	−0.0042 (5)	0.0014 (5)
O32	0.0116 (6)	0.0181 (6)	0.0174 (7)	0.0014 (5)	0.0025 (5)	0.0010 (5)
Na3	0.0128 (3)	0.0131 (3)	0.0162 (4)	−0.0009 (2)	0.0038 (3)	−0.0015 (3)
C21	0.0110 (7)	0.0105 (7)	0.0094 (8)	0.0020 (6)	−0.0017 (6)	−0.0008 (6)
O21	0.0134 (5)	0.0083 (6)	0.0172 (6)	−0.0023 (4)	−0.0010 (5)	−0.0011 (5)
C22	0.0103 (7)	0.0093 (7)	0.0097 (8)	0.0004 (6)	−0.0008 (6)	−0.0013 (6)
O22	0.0144 (5)	0.0106 (6)	0.0124 (6)	−0.0003 (5)	−0.0011 (5)	−0.0059 (5)
C23	0.0111 (7)	0.0068 (7)	0.0121 (8)	0.0000 (5)	−0.0017 (6)	−0.0001 (6)
O23	0.0158 (6)	0.0078 (5)	0.0172 (7)	0.0020 (4)	−0.0090 (5)	−0.0013 (5)
C24	0.0088 (6)	0.0070 (7)	0.0122 (8)	0.0007 (6)	0.0001 (6)	0.0002 (6)
O24	0.0083 (5)	0.0176 (6)	0.0208 (7)	0.0008 (5)	0.0023 (5)	−0.0004 (5)
C25	0.0084 (6)	0.0083 (7)	0.0112 (8)	−0.0014 (6)	0.0004 (6)	0.0005 (6)
O25	0.0087 (5)	0.0105 (6)	0.0160 (6)	−0.0007 (4)	−0.0002 (4)	−0.0021 (5)
C26	0.0158 (7)	0.0147 (8)	0.0115 (8)	0.0018 (6)	−0.0016 (6)	−0.0016 (6)
O26	0.0220 (6)	0.0181 (6)	0.0127 (6)	0.0012 (5)	−0.0083 (5)	−0.0061 (5)
S3	0.01138 (16)	0.01143 (17)	0.00994 (19)	0.00368 (14)	−0.00212 (15)	−0.00099 (16)
O27	0.0182 (5)	0.0174 (6)	0.0112 (6)	0.0044 (5)	−0.0012 (5)	−0.0022 (5)
O28	0.0118 (5)	0.0207 (6)	0.0200 (7)	−0.0007 (4)	−0.0050 (4)	0.0019 (5)
O29	0.0243 (6)	0.0182 (6)	0.0133 (6)	0.0117 (5)	−0.0026 (5)	0.0001 (5)
O33	0.0161 (6)	0.0170 (7)	0.0638 (11)	−0.0002 (5)	−0.0121 (7)	0.0021 (7)

### Geometric parameters (Å, º)

**Table:**

Na1—O31	2.3524 (14)	O13—H13O	0.768 (18)
Na1—O9^i^	2.3577 (12)	C14—O14	1.4379 (17)
Na1—O17	2.4017 (12)	C14—C15	1.540 (2)
Na1—O18^i^	2.4031 (13)	C14—H14	0.98
Na1—O11	2.4570 (14)	O14—H14O	0.77 (2)
Na1—O12	2.4958 (13)	C15—O15	1.4406 (18)
Na1—C11	3.1258 (16)	C15—C16	1.510 (2)
Na1—S2^i^	3.2410 (7)	C15—H15	0.98
Na1—S2	3.3303 (7)	O15—H15O	0.805 (19)
Na1—H31B	2.58 (2)	C16—O16	1.4275 (18)
C1—O1	1.4083 (18)	C16—H16A	0.97
C1—C2	1.525 (2)	C16—H16B	0.97
C1—S1	1.8172 (15)	O16—H16O	0.74 (2)
C1—Na2	3.1275 (17)	S2—O17	1.4479 (11)
C1—H1	0.98	S2—O18	1.4591 (11)
O1—Na2	2.4325 (13)	S2—O19	1.4640 (10)
O1—H1O	0.81 (2)	S2—Na1^iii^	3.2410 (7)
C2—O2	1.4335 (18)	O18—Na1^iii^	2.4031 (13)
C2—C3	1.527 (2)	O19—Na2^iii^	2.3857 (12)
C2—H2	0.98	O32—H32A	0.89 (2)
O2—Na2	2.5483 (13)	O32—H32B	0.741 (18)
O2—H2O	0.770 (17)	Na3—O33	2.2778 (14)
C3—O3	1.4311 (18)	Na3—O8^iv^	2.3669 (12)
C3—C4	1.527 (2)	Na3—O21	2.3824 (13)
C3—H3	0.98	Na3—O29^iv^	2.3955 (12)
O3—H3O	0.813 (19)	Na3—O22	2.4329 (13)
C4—O4	1.4336 (17)	Na3—O27	2.4874 (12)
C4—C5	1.540 (2)	Na3—C21	3.0959 (16)
C4—H4	0.98	Na3—S3	3.3744 (7)
O4—H4O	0.84 (2)	Na3—H33A	2.678 (19)
C5—O5	1.4386 (17)	C21—O21	1.4106 (17)
C5—C6	1.506 (2)	C21—C22	1.523 (2)
C5—H5	0.98	C21—S3	1.8166 (14)
O5—H5O	0.79 (2)	C21—H21	0.98
C6—O6	1.4290 (19)	O21—H21O	0.84 (2)
C6—H6A	0.97	C22—O22	1.4319 (18)
C6—H6B	0.97	C22—C23	1.521 (2)
O6—H6O	0.76 (2)	C22—H22	0.98
S1—O7	1.4484 (12)	O22—H22O	0.765 (18)
S1—O8	1.4595 (10)	C23—O23	1.4339 (17)
S1—O9	1.4608 (10)	C23—C24	1.524 (2)
S1—Na2	3.3760 (7)	C23—H23	0.98
O7—Na2	2.4452 (12)	O23—H23O	0.83 (2)
O8—Na3^ii^	2.3669 (12)	C24—O24	1.4374 (17)
O9—Na1^iii^	2.3577 (12)	C24—C25	1.530 (2)
O31—H31A	0.700 (19)	C24—H24	0.98
O31—H31B	0.83 (2)	O24—H24O	0.76 (2)
Na2—O32	2.3812 (14)	C25—O25	1.4449 (17)
Na2—O19^i^	2.3857 (12)	C25—C26	1.508 (2)
Na2—O28^iv^	2.4239 (13)	C25—H25	0.98
Na2—O27^iv^	2.7802 (12)	O25—H25O	0.777 (19)
Na2—S3^iv^	3.1271 (7)	C26—O26	1.4280 (19)
C11—O11	1.4103 (17)	C26—H26A	0.97
C11—C12	1.522 (2)	C26—H26B	0.97
C11—S2	1.8183 (15)	O26—H26O	0.76 (2)
C11—H11	0.98	S3—O29	1.4526 (11)
O11—H11O	0.80 (2)	S3—O27	1.4621 (11)
C12—O12	1.4363 (18)	S3—O28	1.4666 (11)
C12—C13	1.532 (2)	S3—Na2^ii^	3.1271 (7)
C12—H12	0.98	O27—Na2^ii^	2.7802 (12)
O12—H12O	0.81 (2)	O28—Na2^ii^	2.4239 (13)
C13—O13	1.4351 (18)	O29—Na3^ii^	2.3955 (12)
C13—C14	1.524 (2)	O33—H33A	0.833 (19)
C13—H13	0.98	O33—H33B	0.80 (3)
			
O31—Na1—O9^i^	92.30 (5)	Na1—O11—H11O	137.9 (17)
O31—Na1—O17	147.47 (5)	O12—C12—C11	110.16 (12)
O9^i^—Na1—O17	97.93 (4)	O12—C12—C13	105.85 (11)
O31—Na1—O18^i^	117.27 (5)	C11—C12—C13	112.61 (12)
O9^i^—Na1—O18^i^	87.29 (4)	O12—C12—H12	109.4
O17—Na1—O18^i^	94.09 (4)	C11—C12—H12	109.4
O31—Na1—O11	75.30 (4)	C13—C12—H12	109.4
O9^i^—Na1—O11	107.86 (4)	C12—O12—Na1	111.64 (8)
O17—Na1—O11	72.17 (4)	C12—O12—H12O	105.7 (14)
O18^i^—Na1—O11	160.51 (4)	Na1—O12—H12O	116.0 (14)
O31—Na1—O12	95.18 (5)	O13—C13—C14	109.46 (12)
O9^i^—Na1—O12	171.33 (5)	O13—C13—C12	105.62 (12)
O17—Na1—O12	73.40 (4)	C14—C13—C12	114.68 (12)
O18^i^—Na1—O12	93.14 (4)	O13—C13—H13	109
O11—Na1—O12	70.02 (4)	C14—C13—H13	109
O31—Na1—C11	94.06 (5)	C12—C13—H13	109
O9^i^—Na1—C11	125.53 (4)	C13—O13—H13O	106.6 (14)
O17—Na1—C11	55.10 (4)	O14—C14—C13	109.43 (12)
O18^i^—Na1—C11	134.63 (4)	O14—C14—C15	106.83 (12)
O11—Na1—C11	25.89 (4)	C13—C14—C15	112.08 (12)
O12—Na1—C11	49.59 (4)	O14—C14—H14	109.5
O31—Na1—S2^i^	92.86 (4)	C13—C14—H14	109.5
O9^i^—Na1—S2^i^	92.08 (3)	C15—C14—H14	109.5
O17—Na1—S2^i^	117.38 (4)	C14—O14—H14O	107.3 (15)
O18^i^—Na1—S2^i^	24.72 (3)	O15—C15—C16	107.50 (12)
O11—Na1—S2^i^	156.93 (4)	O15—C15—C14	111.01 (13)
O12—Na1—S2^i^	91.90 (3)	C16—C15—C14	113.51 (13)
C11—Na1—S2^i^	141.33 (3)	O15—C15—H15	108.2
O31—Na1—S2	125.47 (4)	C16—C15—H15	108.2
O9^i^—Na1—S2	110.06 (3)	C14—C15—H15	108.2
O17—Na1—S2	22.65 (3)	C15—O15—H15O	105.4 (13)
O18^i^—Na1—S2	113.01 (3)	O16—C16—C15	109.55 (13)
O11—Na1—S2	50.87 (3)	O16—C16—H16A	109.8
O12—Na1—S2	61.86 (3)	C15—C16—H16A	109.8
C11—Na1—S2	32.52 (3)	O16—C16—H16B	109.8
S2^i^—Na1—S2	133.06 (2)	C15—C16—H16B	109.8
O31—Na1—H31B	18.6 (6)	H16A—C16—H16B	108.2
O9^i^—Na1—H31B	88.4 (5)	C16—O16—H16O	108.1 (17)
O17—Na1—H31B	130.7 (6)	O17—S2—O18	112.27 (7)
O18^i^—Na1—H31B	135.2 (6)	O17—S2—O19	113.09 (7)
O11—Na1—H31B	59.5 (6)	O18—S2—O19	112.94 (6)
O12—Na1—H31B	97.3 (5)	O17—S2—C11	107.07 (6)
C11—Na1—H31B	81.6 (6)	O18—S2—C11	106.19 (7)
S2^i^—Na1—H31B	111.1 (6)	O19—S2—C11	104.54 (7)
S2—Na1—H31B	110.4 (6)	O17—S2—Na1^iii^	68.83 (5)
O1—C1—C2	108.85 (12)	O18—S2—Na1^iii^	43.52 (5)
O1—C1—S1	107.98 (10)	O19—S2—Na1^iii^	130.66 (5)
C2—C1—S1	112.08 (10)	C11—S2—Na1^iii^	122.49 (5)
O1—C1—Na2	48.41 (7)	O17—S2—Na1	39.71 (5)
C2—C1—Na2	83.02 (9)	O18—S2—Na1	127.05 (5)
S1—C1—Na2	81.47 (6)	O19—S2—Na1	119.56 (4)
O1—C1—H1	109.3	C11—S2—Na1	67.54 (5)
C2—C1—H1	109.3	Na1^iii^—S2—Na1	93.415 (13)
S1—C1—H1	109.3	S2—O17—Na1	117.64 (6)
Na2—C1—H1	157.7	S2—O18—Na1^iii^	111.76 (6)
C1—O1—Na2	105.93 (9)	S2—O19—Na2^iii^	135.63 (7)
C1—O1—H1O	109.7 (15)	Na2—O32—H32A	101.7 (13)
Na2—O1—H1O	132.2 (15)	Na2—O32—H32B	119.5 (13)
O2—C2—C1	111.00 (13)	H32A—O32—H32B	105.9 (18)
O2—C2—C3	104.85 (12)	O33—Na3—O8^iv^	92.29 (5)
C1—C2—C3	111.04 (12)	O33—Na3—O21	91.45 (5)
O2—C2—H2	109.9	O8^iv^—Na3—O21	157.31 (5)
C1—C2—H2	109.9	O33—Na3—O29^iv^	75.20 (5)
C3—C2—H2	109.9	O8^iv^—Na3—O29^iv^	99.47 (4)
C2—O2—Na2	109.17 (8)	O21—Na3—O29^iv^	103.12 (5)
C2—O2—H2O	104.4 (13)	O33—Na3—O22	115.83 (6)
Na2—O2—H2O	99.8 (14)	O8^iv^—Na3—O22	87.82 (4)
O3—C3—C2	106.65 (12)	O21—Na3—O22	70.45 (4)
O3—C3—C4	109.67 (12)	O29^iv^—Na3—O22	166.73 (5)
C2—C3—C4	115.14 (12)	O33—Na3—O27	156.36 (5)
O3—C3—H3	108.4	O8^iv^—Na3—O27	109.72 (4)
C2—C3—H3	108.4	O21—Na3—O27	71.67 (4)
C4—C3—H3	108.4	O29^iv^—Na3—O27	92.24 (4)
C3—O3—H3O	107.4 (13)	O22—Na3—O27	74.80 (4)
O4—C4—C3	109.37 (12)	O33—Na3—C21	113.71 (5)
O4—C4—C5	109.38 (12)	O8^iv^—Na3—C21	136.36 (4)
C3—C4—C5	110.95 (12)	O21—Na3—C21	25.89 (4)
O4—C4—H4	109	O29^iv^—Na3—C21	120.16 (4)
C3—C4—H4	109	O22—Na3—C21	49.86 (4)
C5—C4—H4	109	O27—Na3—C21	55.38 (4)
C4—O4—H4O	108.6 (14)	O33—Na3—S3	142.11 (5)
O5—C5—C6	106.02 (12)	O8^iv^—Na3—S3	123.93 (3)
O5—C5—C4	109.30 (12)	O21—Na3—S3	50.94 (3)
C6—C5—C4	113.00 (12)	O29^iv^—Na3—S3	105.82 (3)
O5—C5—H5	109.5	O22—Na3—S3	61.01 (3)
C6—C5—H5	109.5	O27—Na3—S3	23.14 (3)
C4—C5—H5	109.5	C21—Na3—S3	32.25 (3)
C5—O5—H5O	118.7 (18)	O33—Na3—H33A	17.0 (4)
O6—C6—C5	107.26 (12)	O8^iv^—Na3—H33A	106.4 (4)
O6—C6—H6A	110.3	O21—Na3—H33A	81.9 (4)
C5—C6—H6A	110.3	O29^iv^—Na3—H33A	64.2 (4)
O6—C6—H6B	110.3	O22—Na3—H33A	124.5 (4)
C5—C6—H6B	110.3	O27—Na3—H33A	139.6 (4)
H6A—C6—H6B	108.5	C21—Na3—H33A	106.9 (4)
C6—O6—H6O	111.3 (16)	S3—Na3—H33A	129.6 (4)
O7—S1—O8	113.15 (7)	O21—C21—C22	108.97 (12)
O7—S1—O9	113.05 (7)	O21—C21—S3	108.68 (10)
O8—S1—O9	112.85 (6)	C22—C21—S3	110.29 (10)
O7—S1—C1	106.11 (7)	O21—C21—Na3	47.52 (7)
O8—S1—C1	105.79 (7)	C22—C21—Na3	82.72 (8)
O9—S1—C1	105.02 (7)	S3—C21—Na3	82.35 (6)
O7—S1—Na2	39.78 (5)	O21—C21—H21	109.6
O8—S1—Na2	125.31 (5)	C22—C21—H21	109.6
O9—S1—Na2	121.54 (4)	S3—C21—H21	109.6
C1—S1—Na2	66.37 (5)	Na3—C21—H21	157.1
S1—O7—Na2	117.95 (7)	C21—O21—Na3	106.59 (9)
S1—O8—Na3^ii^	133.20 (7)	C21—O21—H21O	112.5 (14)
S1—O9—Na1^iii^	134.70 (7)	Na3—O21—H21O	129.3 (14)
Na1—O31—H31A	124.0 (17)	O22—C22—C23	106.87 (12)
Na1—O31—H31B	96.7 (17)	O22—C22—C21	109.05 (12)
H31A—O31—H31B	110 (2)	C23—C22—C21	112.26 (12)
O32—Na2—O19^i^	90.30 (5)	O22—C22—H22	109.5
O32—Na2—O28^iv^	131.93 (5)	C23—C22—H22	109.5
O19^i^—Na2—O28^iv^	83.62 (4)	C21—C22—H22	109.5
O32—Na2—O1	77.37 (4)	C22—O22—Na3	113.14 (9)
O19^i^—Na2—O1	111.11 (4)	C22—O22—H22O	104.5 (14)
O28^iv^—Na2—O1	148.51 (4)	Na3—O22—H22O	125.0 (15)
O32—Na2—O7	145.58 (5)	O23—C23—C22	105.85 (12)
O19^i^—Na2—O7	88.56 (4)	O23—C23—C24	110.29 (13)
O28^iv^—Na2—O7	82.09 (4)	C22—C23—C24	114.34 (12)
O1—Na2—O7	71.05 (4)	O23—C23—H23	108.7
O32—Na2—O2	109.30 (5)	C22—C23—H23	108.7
O19^i^—Na2—O2	159.82 (5)	C24—C23—H23	108.7
O28^iv^—Na2—O2	86.53 (4)	C23—O23—H23O	108.9 (15)
O1—Na2—O2	70.22 (4)	O24—C24—C23	109.66 (12)
O7—Na2—O2	72.63 (4)	O24—C24—C25	108.04 (12)
O32—Na2—O27^iv^	81.71 (4)	C23—C24—C25	111.46 (12)
O19^i^—Na2—O27^iv^	105.82 (4)	O24—C24—H24	109.2
O28^iv^—Na2—O27^iv^	54.91 (4)	C23—C24—H24	109.2
O1—Na2—O27^iv^	137.24 (4)	C25—C24—H24	109.2
O7—Na2—O27^iv^	131.45 (4)	C24—O24—H24O	106.5 (15)
O2—Na2—O27^iv^	82.44 (4)	O25—C25—C26	106.73 (12)
O32—Na2—S3^iv^	107.62 (4)	O25—C25—C24	109.19 (12)
O19^i^—Na2—S3^iv^	95.18 (3)	C26—C25—C24	112.65 (12)
O28^iv^—Na2—S3^iv^	27.03 (3)	O25—C25—H25	109.4
O1—Na2—S3^iv^	153.37 (4)	C26—C25—H25	109.4
O7—Na2—S3^iv^	106.74 (3)	C24—C25—H25	109.4
O2—Na2—S3^iv^	83.68 (3)	C25—O25—H25O	105.1 (14)
O27^iv^—Na2—S3^iv^	27.87 (2)	O26—C26—C25	107.57 (12)
O32—Na2—C1	99.46 (5)	O26—C26—H26A	110.2
O19^i^—Na2—C1	124.12 (4)	C25—C26—H26A	110.2
O28^iv^—Na2—C1	123.19 (4)	O26—C26—H26B	110.2
O1—Na2—C1	25.66 (4)	C25—C26—H26B	110.2
O7—Na2—C1	54.42 (4)	H26A—C26—H26B	108.5
O2—Na2—C1	49.61 (4)	C26—O26—H26O	108.7 (18)
O27^iv^—Na2—C1	129.96 (4)	O29—S3—O27	113.12 (7)
S3^iv^—Na2—C1	131.89 (3)	O29—S3—O28	112.95 (7)
O32—Na2—S1	127.73 (4)	O27—S3—O28	111.44 (7)
O19^i^—Na2—S1	103.72 (3)	O29—S3—C21	106.35 (7)
O28^iv^—Na2—S1	99.89 (3)	O27—S3—C21	107.35 (7)
O1—Na2—S1	50.49 (3)	O28—S3—C21	104.98 (7)
O7—Na2—S1	22.27 (3)	O29—S3—Na2^ii^	133.17 (5)
O2—Na2—S1	60.73 (3)	O27—S3—Na2^ii^	62.75 (5)
O27^iv^—Na2—S1	137.71 (3)	O28—S3—Na2^ii^	48.70 (5)
S3^iv^—Na2—S1	120.50 (2)	C21—S3—Na2^ii^	119.59 (5)
C1—Na2—S1	32.16 (3)	O29—S3—Na3	126.14 (5)
O11—C11—C12	109.48 (11)	O27—S3—Na3	41.97 (4)
O11—C11—S2	107.06 (10)	O28—S3—Na3	120.66 (5)
C12—C11—S2	112.14 (10)	C21—S3—Na3	65.41 (5)
O11—C11—Na1	49.53 (7)	Na2^ii^—S3—Na3	83.765 (17)
C12—C11—Na1	83.05 (8)	S3—O27—Na3	114.89 (6)
S2—C11—Na1	79.94 (5)	S3—O27—Na2^ii^	89.38 (5)
O11—C11—H11	109.4	Na3—O27—Na2^ii^	111.00 (4)
C12—C11—H11	109.4	S3—O28—Na2^ii^	104.27 (6)
S2—C11—H11	109.4	S3—O29—Na3^ii^	137.50 (7)
Na1—C11—H11	158.8	Na3—O33—H33A	109.9 (13)
C11—O11—Na1	104.58 (9)	Na3—O33—H33B	142.1 (18)
C11—O11—H11O	105.5 (16)	H33A—O33—H33B	100 (2)
			
C2—C1—O1—Na2	−61.66 (12)	C12—C11—S2—O19	−165.44 (10)
S1—C1—O1—Na2	60.24 (9)	Na1—C11—S2—O19	116.38 (5)
O1—C1—C2—O2	63.89 (15)	O11—C11—S2—Na1^iii^	−121.15 (8)
S1—C1—C2—O2	−55.48 (14)	C12—C11—S2—Na1^iii^	−1.05 (13)
Na2—C1—C2—O2	22.35 (10)	Na1—C11—S2—Na1^iii^	−79.23 (4)
O1—C1—C2—C3	−52.32 (16)	O11—C11—S2—Na1	−41.92 (8)
S1—C1—C2—C3	−171.69 (10)	C12—C11—S2—Na1	78.18 (10)
Na2—C1—C2—C3	−93.86 (11)	O31—Na1—S2—O17	−168.66 (9)
C1—C2—O2—Na2	−29.37 (13)	O9^i^—Na1—S2—O17	−60.44 (8)
C3—C2—O2—Na2	90.61 (11)	O18^i^—Na1—S2—O17	35.31 (9)
O2—C2—C3—O3	64.02 (14)	O11—Na1—S2—O17	−157.56 (9)
C1—C2—C3—O3	−176.03 (12)	O12—Na1—S2—O17	116.13 (8)
O2—C2—C3—C4	−174.09 (12)	C11—Na1—S2—O17	174.23 (9)
C1—C2—C3—C4	−54.14 (17)	S2^i^—Na1—S2—O17	52.77 (8)
O3—C3—C4—O4	57.66 (15)	O31—Na1—S2—O18	110.89 (8)
C2—C3—C4—O4	−62.60 (16)	O9^i^—Na1—S2—O18	−140.89 (7)
O3—C3—C4—C5	−63.10 (15)	O17—Na1—S2—O18	−80.45 (10)
C2—C3—C4—C5	176.65 (12)	O18^i^—Na1—S2—O18	−45.14 (9)
O4—C4—C5—O5	179.08 (12)	O11—Na1—S2—O18	121.99 (7)
C3—C4—C5—O5	−60.17 (15)	O12—Na1—S2—O18	35.68 (7)
O4—C4—C5—C6	61.28 (16)	C11—Na1—S2—O18	93.79 (8)
C3—C4—C5—C6	−177.98 (12)	S2^i^—Na1—S2—O18	−27.68 (7)
O5—C5—C6—O6	56.17 (15)	O31—Na1—S2—O19	−77.36 (7)
C4—C5—C6—O6	175.88 (12)	O9^i^—Na1—S2—O19	30.86 (7)
O1—C1—S1—O7	−42.71 (11)	O17—Na1—S2—O19	91.31 (10)
C2—C1—S1—O7	77.17 (11)	O18^i^—Na1—S2—O19	126.62 (6)
Na2—C1—S1—O7	−1.68 (6)	O11—Na1—S2—O19	−66.26 (7)
O1—C1—S1—O8	−163.17 (9)	O12—Na1—S2—O19	−152.56 (7)
C2—C1—S1—O8	−43.29 (12)	C11—Na1—S2—O19	−94.46 (7)
Na2—C1—S1—O8	−122.13 (5)	S2^i^—Na1—S2—O19	144.08 (6)
O1—C1—S1—O9	77.25 (11)	O31—Na1—S2—C11	17.10 (7)
C2—C1—S1—O9	−162.87 (10)	O9^i^—Na1—S2—C11	125.32 (6)
Na2—C1—S1—O9	118.28 (5)	O17—Na1—S2—C11	−174.23 (9)
O1—C1—S1—Na2	−41.03 (8)	O18^i^—Na1—S2—C11	−138.92 (6)
C2—C1—S1—Na2	78.85 (10)	O11—Na1—S2—C11	28.20 (6)
O8—S1—O7—Na2	117.97 (7)	O12—Na1—S2—C11	−58.10 (6)
O9—S1—O7—Na2	−112.18 (7)	S2^i^—Na1—S2—C11	−121.46 (6)
C1—S1—O7—Na2	2.40 (8)	O31—Na1—S2—Na1^iii^	141.00 (5)
O7—S1—O8—Na3^ii^	−2.09 (11)	O9^i^—Na1—S2—Na1^iii^	−110.78 (4)
O9—S1—O8—Na3^ii^	−132.04 (8)	O17—Na1—S2—Na1^iii^	−50.34 (8)
C1—S1—O8—Na3^ii^	113.67 (9)	O18^i^—Na1—S2—Na1^iii^	−15.03 (3)
Na2—S1—O8—Na3^ii^	41.74 (11)	O11—Na1—S2—Na1^iii^	152.10 (4)
O7—S1—O9—Na1^iii^	−54.52 (10)	O12—Na1—S2—Na1^iii^	65.79 (3)
O8—S1—O9—Na1^iii^	75.48 (10)	C11—Na1—S2—Na1^iii^	123.89 (5)
C1—S1—O9—Na1^iii^	−169.76 (8)	S2^i^—Na1—S2—Na1^iii^	2.430 (15)
Na2—S1—O9—Na1^iii^	−98.56 (8)	O18—S2—O17—Na1	121.74 (7)
C1—O1—Na2—O32	148.82 (9)	O19—S2—O17—Na1	−109.03 (7)
C1—O1—Na2—O19^i^	−125.81 (8)	C11—S2—O17—Na1	5.57 (9)
C1—O1—Na2—O28^iv^	−12.09 (14)	Na1^iii^—S2—O17—Na1	124.50 (7)
C1—O1—Na2—O7	−45.08 (8)	O31—Na1—O17—S2	17.32 (14)
C1—O1—Na2—O2	32.68 (8)	O9^i^—Na1—O17—S2	124.41 (8)
C1—O1—Na2—O27^iv^	86.20 (10)	O18^i^—Na1—O17—S2	−147.76 (8)
C1—O1—Na2—S3^iv^	44.62 (13)	O11—Na1—O17—S2	18.12 (7)
C1—O1—Na2—S1	−35.18 (7)	O12—Na1—O17—S2	−55.70 (7)
S1—O7—Na2—O32	45.00 (12)	C11—Na1—O17—S2	−3.78 (6)
S1—O7—Na2—O19^i^	133.42 (7)	S2^i^—Na1—O17—S2	−139.07 (6)
S1—O7—Na2—O28^iv^	−142.81 (7)	O17—S2—O18—Na1^iii^	3.74 (9)
S1—O7—Na2—O1	20.50 (7)	O19—S2—O18—Na1^iii^	−125.57 (7)
S1—O7—Na2—O2	−53.99 (7)	C11—S2—O18—Na1^iii^	120.43 (7)
S1—O7—Na2—O27^iv^	−116.60 (7)	Na1—S2—O18—Na1^iii^	46.64 (8)
S1—O7—Na2—S3^iv^	−131.60 (6)	O17—S2—O19—Na2^iii^	−43.70 (10)
S1—O7—Na2—C1	−1.65 (6)	O18—S2—O19—Na2^iii^	85.18 (10)
C2—O2—Na2—O32	−68.99 (10)	C11—S2—O19—Na2^iii^	−159.82 (8)
C2—O2—Na2—O19^i^	96.70 (15)	Na1^iii^—S2—O19—Na2^iii^	37.59 (11)
C2—O2—Na2—O28^iv^	157.53 (9)	Na1—S2—O19—Na2^iii^	−87.68 (8)
C2—O2—Na2—O1	−0.83 (8)	O33—Na3—C21—O21	−32.71 (10)
C2—O2—Na2—O7	74.74 (9)	O8^iv^—Na3—C21—O21	−154.29 (9)
C2—O2—Na2—O27^iv^	−147.42 (9)	O29^iv^—Na3—C21—O21	53.42 (10)
C2—O2—Na2—S3^iv^	−175.48 (9)	O22—Na3—C21—O21	−137.32 (10)
C2—O2—Na2—C1	17.04 (8)	O27—Na3—C21—O21	123.16 (10)
C2—O2—Na2—S1	54.17 (8)	S3—Na3—C21—O21	124.14 (11)
O1—C1—Na2—O32	−30.81 (9)	O33—Na3—C21—C22	91.45 (9)
C2—C1—Na2—O32	92.14 (8)	O8^iv^—Na3—C21—C22	−30.14 (10)
S1—C1—Na2—O32	−154.20 (5)	O21—Na3—C21—C22	124.15 (12)
O1—C1—Na2—O19^i^	66.05 (10)	O29^iv^—Na3—C21—C22	177.58 (8)
C2—C1—Na2—O19^i^	−171.00 (8)	O22—Na3—C21—C22	−13.16 (7)
S1—C1—Na2—O19^i^	−57.34 (7)	O27—Na3—C21—C22	−112.68 (9)
O1—C1—Na2—O28^iv^	172.48 (9)	S3—Na3—C21—C22	−111.71 (10)
C2—C1—Na2—O28^iv^	−64.56 (9)	O33—Na3—C21—S3	−156.84 (6)
S1—C1—Na2—O28^iv^	49.09 (7)	O8^iv^—Na3—C21—S3	81.57 (8)
C2—C1—Na2—O1	122.95 (13)	O21—Na3—C21—S3	−124.14 (11)
S1—C1—Na2—O1	−123.39 (11)	O29^iv^—Na3—C21—S3	−70.71 (6)
O1—C1—Na2—O7	124.56 (10)	O22—Na3—C21—S3	98.55 (6)
C2—C1—Na2—O7	−112.48 (9)	O27—Na3—C21—S3	−0.97 (4)
S1—C1—Na2—O7	1.17 (4)	C22—C21—O21—Na3	−60.23 (12)
O1—C1—Na2—O2	−138.15 (10)	S3—C21—O21—Na3	59.99 (10)
C2—C1—Na2—O2	−15.20 (7)	O33—Na3—O21—C21	150.34 (9)
S1—C1—Na2—O2	98.46 (6)	O8^iv^—Na3—O21—C21	50.90 (16)
O1—C1—Na2—O27^iv^	−117.89 (9)	O29^iv^—Na3—O21—C21	−134.53 (9)
C2—C1—Na2—O27^iv^	5.06 (10)	O22—Na3—O21—C21	33.36 (8)
S1—C1—Na2—O27^iv^	118.72 (6)	O27—Na3—O21—C21	−46.53 (8)
O1—C1—Na2—S3^iv^	−154.98 (8)	S3—Na3—O21—C21	−34.66 (7)
C2—C1—Na2—S3^iv^	−32.03 (10)	O21—C21—C22—O22	58.43 (15)
S1—C1—Na2—S3^iv^	81.63 (6)	S3—C21—C22—O22	−60.79 (13)
O1—C1—Na2—S1	123.39 (11)	Na3—C21—C22—O22	18.23 (10)
C2—C1—Na2—S1	−113.66 (10)	O21—C21—C22—C23	−59.80 (16)
O7—S1—Na2—O32	−149.65 (9)	S3—C21—C22—C23	−179.02 (10)
O8—S1—Na2—O32	125.98 (7)	Na3—C21—C22—C23	−100.00 (11)
O9—S1—Na2—O32	−60.76 (8)	C23—C22—O22—Na3	96.13 (11)
C1—S1—Na2—O32	32.87 (7)	C21—C22—O22—Na3	−25.44 (13)
O7—S1—Na2—O19^i^	−48.36 (8)	O33—Na3—O22—C22	−85.00 (10)
O8—S1—Na2—O19^i^	−132.74 (7)	O8^iv^—Na3—O22—C22	−176.49 (9)
O9—S1—Na2—O19^i^	40.52 (7)	O21—Na3—O22—C22	−3.17 (9)
C1—S1—Na2—O19^i^	134.16 (6)	O29^iv^—Na3—O22—C22	59.7 (2)
O7—S1—Na2—O28^iv^	37.42 (7)	O27—Na3—O22—C22	72.39 (9)
O8—S1—Na2—O28^iv^	−46.95 (7)	C21—Na3—O22—C22	15.14 (8)
O9—S1—Na2—O28^iv^	126.31 (6)	S3—Na3—O22—C22	52.24 (8)
C1—S1—Na2—O28^iv^	−140.06 (6)	O22—C22—C23—O23	65.17 (15)
O7—S1—Na2—O1	−154.58 (8)	C21—C22—C23—O23	−175.32 (12)
O8—S1—Na2—O1	121.05 (7)	O22—C22—C23—C24	−173.24 (12)
O9—S1—Na2—O1	−65.69 (7)	C21—C22—C23—C24	−53.72 (17)
C1—S1—Na2—O1	27.94 (6)	O23—C23—C24—O24	60.83 (15)
O8—S1—Na2—O7	−84.38 (9)	C22—C23—C24—O24	−58.29 (16)
O9—S1—Na2—O7	88.89 (9)	O23—C23—C24—C25	−58.75 (15)
C1—S1—Na2—O7	−177.48 (8)	C22—C23—C24—C25	−177.88 (12)
O7—S1—Na2—O2	117.75 (8)	O24—C24—C25—O25	−167.35 (12)
O8—S1—Na2—O2	33.37 (7)	C23—C24—C25—O25	−46.81 (16)
O9—S1—Na2—O2	−153.36 (7)	O24—C24—C25—C26	74.26 (15)
C1—S1—Na2—O2	−59.73 (6)	C23—C24—C25—C26	−165.20 (13)
O7—S1—Na2—O27^iv^	84.91 (8)	O25—C25—C26—O26	55.48 (15)
O8—S1—Na2—O27^iv^	0.54 (8)	C24—C25—C26—O26	175.30 (12)
O9—S1—Na2—O27^iv^	173.80 (7)	O21—C21—S3—O29	82.67 (11)
C1—S1—Na2—O27^iv^	−92.56 (7)	C22—C21—S3—O29	−157.93 (10)
O7—S1—Na2—S3^iv^	56.22 (7)	Na3—C21—S3—O29	122.79 (6)
O8—S1—Na2—S3^iv^	−28.16 (7)	O21—C21—S3—O27	−38.69 (11)
O9—S1—Na2—S3^iv^	145.10 (6)	C22—C21—S3—O27	80.71 (11)
C1—S1—Na2—S3^iv^	−121.26 (6)	Na3—C21—S3—O27	1.43 (6)
O7—S1—Na2—C1	177.48 (8)	O21—C21—S3—O28	−157.38 (10)
O8—S1—Na2—C1	93.10 (8)	C22—C21—S3—O28	−37.98 (12)
O9—S1—Na2—C1	−93.63 (8)	Na3—C21—S3—O28	−117.26 (5)
O31—Na1—C11—O11	−43.20 (9)	O21—C21—S3—Na2^ii^	−106.71 (9)
O9^i^—Na1—C11—O11	52.55 (10)	C22—C21—S3—Na2^ii^	12.69 (12)
O17—Na1—C11—O11	125.61 (10)	Na3—C21—S3—Na2^ii^	−66.59 (5)
O18^i^—Na1—C11—O11	−178.89 (9)	O21—C21—S3—Na3	−40.12 (8)
O12—Na1—C11—O11	−136.56 (10)	C22—C21—S3—Na3	79.28 (10)
S2^i^—Na1—C11—O11	−142.92 (8)	O33—Na3—S3—O29	−56.93 (10)
S2—Na1—C11—O11	122.90 (10)	O8^iv^—Na3—S3—O29	142.54 (7)
O31—Na1—C11—C12	79.87 (8)	O21—Na3—S3—O29	−65.08 (7)
O9^i^—Na1—C11—C12	175.62 (8)	O29^iv^—Na3—S3—O29	29.16 (9)
O17—Na1—C11—C12	−111.32 (9)	O22—Na3—S3—O29	−152.62 (7)
O18^i^—Na1—C11—C12	−55.82 (10)	O27—Na3—S3—O29	85.14 (9)
O11—Na1—C11—C12	123.07 (12)	C21—Na3—S3—O29	−92.82 (8)
O12—Na1—C11—C12	−13.48 (7)	O33—Na3—S3—O27	−142.06 (10)
S2^i^—Na1—C11—C12	−19.85 (10)	O8^iv^—Na3—S3—O27	57.40 (8)
S2—Na1—C11—C12	−114.03 (10)	O21—Na3—S3—O27	−150.22 (8)
O31—Na1—C11—S2	−166.11 (5)	O29^iv^—Na3—S3—O27	−55.98 (7)
O9^i^—Na1—C11—S2	−70.35 (7)	O22—Na3—S3—O27	122.24 (7)
O17—Na1—C11—S2	2.70 (4)	C21—Na3—S3—O27	−177.96 (9)
O18^i^—Na1—C11—S2	58.20 (8)	O33—Na3—S3—O28	129.29 (9)
O11—Na1—C11—S2	−122.90 (10)	O8^iv^—Na3—S3—O28	−31.25 (8)
O12—Na1—C11—S2	100.54 (6)	O21—Na3—S3—O28	121.14 (7)
S2^i^—Na1—C11—S2	94.18 (6)	O29^iv^—Na3—S3—O28	−144.62 (6)
C12—C11—O11—Na1	−61.93 (12)	O22—Na3—S3—O28	33.60 (7)
S2—C11—O11—Na1	59.85 (9)	O27—Na3—S3—O28	−88.65 (9)
O31—Na1—O11—C11	135.09 (9)	C21—Na3—S3—O28	93.39 (8)
O9^i^—Na1—O11—C11	−137.25 (8)	O33—Na3—S3—C21	35.89 (9)
O17—Na1—O11—C11	−44.46 (8)	O8^iv^—Na3—S3—C21	−124.64 (7)
O18^i^—Na1—O11—C11	2.36 (19)	O21—Na3—S3—C21	27.74 (6)
O12—Na1—O11—C11	33.86 (8)	O29^iv^—Na3—S3—C21	121.98 (6)
S2^i^—Na1—O11—C11	74.06 (12)	O22—Na3—S3—C21	−59.80 (6)
S2—Na1—O11—C11	−35.58 (7)	O27—Na3—S3—C21	177.96 (9)
O11—C11—C12—O12	61.73 (15)	O33—Na3—S3—Na2^ii^	162.50 (8)
S2—C11—C12—O12	−56.95 (13)	O8^iv^—Na3—S3—Na2^ii^	1.97 (4)
Na1—C11—C12—O12	19.19 (10)	O21—Na3—S3—Na2^ii^	154.35 (4)
O11—C11—C12—C13	−56.18 (16)	O29^iv^—Na3—S3—Na2^ii^	−111.41 (4)
S2—C11—C12—C13	−174.86 (10)	O22—Na3—S3—Na2^ii^	66.81 (4)
Na1—C11—C12—C13	−98.72 (11)	O27—Na3—S3—Na2^ii^	−55.43 (7)
C11—C12—O12—Na1	−26.08 (13)	C21—Na3—S3—Na2^ii^	126.61 (5)
C13—C12—O12—Na1	95.93 (11)	O29—S3—O27—Na3	−118.96 (7)
O31—Na1—O12—C12	−75.63 (9)	O28—S3—O27—Na3	112.49 (7)
O9^i^—Na1—O12—C12	74.0 (3)	C21—S3—O27—Na3	−1.94 (8)
O17—Na1—O12—C12	73.28 (9)	Na2^ii^—S3—O27—Na3	112.96 (6)
O18^i^—Na1—O12—C12	166.62 (9)	O29—S3—O27—Na2^ii^	128.09 (6)
O11—Na1—O12—C12	−3.33 (9)	O28—S3—O27—Na2^ii^	−0.46 (6)
C11—Na1—O12—C12	15.30 (8)	C21—S3—O27—Na2^ii^	−114.90 (6)
S2^i^—Na1—O12—C12	−168.67 (8)	Na3—S3—O27—Na2^ii^	−112.96 (6)
S2—Na1—O12—C12	52.13 (8)	O33—Na3—O27—S3	70.30 (16)
O12—C12—C13—O13	63.56 (14)	O8^iv^—Na3—O27—S3	−132.05 (6)
C11—C12—C13—O13	−176.01 (12)	O21—Na3—O27—S3	23.97 (6)
O12—C12—C13—C14	−175.82 (12)	O29^iv^—Na3—O27—S3	127.06 (7)
C11—C12—C13—C14	−55.40 (16)	O22—Na3—O27—S3	−50.05 (6)
O13—C13—C14—O14	61.49 (15)	C21—Na3—O27—S3	1.32 (6)
C12—C13—C14—O14	−56.99 (16)	O33—Na3—O27—Na2^ii^	169.81 (13)
O13—C13—C14—C15	−56.83 (15)	O8^iv^—Na3—O27—Na2^ii^	−32.54 (6)
C12—C13—C14—C15	−175.31 (12)	O21—Na3—O27—Na2^ii^	123.48 (5)
O14—C14—C15—O15	−164.40 (12)	O29^iv^—Na3—O27—Na2^ii^	−133.43 (5)
C13—C14—C15—O15	−44.55 (16)	O22—Na3—O27—Na2^ii^	49.46 (5)
O14—C14—C15—C16	74.38 (15)	C21—Na3—O27—Na2^ii^	100.83 (6)
C13—C14—C15—C16	−165.77 (12)	S3—Na3—O27—Na2^ii^	99.51 (8)
O15—C15—C16—O16	−57.27 (16)	O29—S3—O28—Na2^ii^	−128.10 (6)
C14—C15—C16—O16	65.90 (16)	O27—S3—O28—Na2^ii^	0.55 (8)
O11—C11—S2—O17	−45.77 (11)	C21—S3—O28—Na2^ii^	116.45 (6)
C12—C11—S2—O17	74.33 (12)	Na3—S3—O28—Na2^ii^	46.46 (6)
Na1—C11—S2—O17	−3.85 (6)	O27—S3—O29—Na3^ii^	−21.01 (12)
O11—C11—S2—O18	−165.90 (9)	O28—S3—O29—Na3^ii^	106.76 (11)
C12—C11—S2—O18	−45.80 (12)	C21—S3—O29—Na3^ii^	−138.61 (10)
Na1—C11—S2—O18	−123.98 (5)	Na2^ii^—S3—O29—Na3^ii^	52.60 (13)
O11—C11—S2—O19	74.46 (10)	Na3—S3—O29—Na3^ii^	−67.44 (11)

Symmetry codes: (i) *x*−1/2, −*y*+1/2, −*z*+1; (ii) *x*+1/2, −*y*+3/2, −*z*+1; (iii) *x*+1/2, −*y*+1/2, −*z*+1; (iv) *x*−1/2, −*y*+3/2, −*z*+1.

### Hydrogen-bond geometry (Å, º)

**Table:**

*D*—H···*A*	*D*—H	H···*A*	*D*···*A*	*D*—H···*A*
O1—H1*O*···O13	0.81 (2)	2.21 (2)	2.8719 (17)	139 (2)
O1—H1*O*···O12	0.81 (2)	2.23 (2)	2.9262 (16)	144 (2)
O2—H2*O*···O33^ii^	0.770 (17)	2.243 (19)	2.927 (2)	148.5 (17)
O3—H3*O*···O15^v^	0.813 (19)	1.989 (19)	2.7862 (15)	166.9 (19)
O4—H4*O*···O24^vi^	0.84 (2)	2.24 (2)	3.0150 (16)	153.0 (19)
O5—H5*O*···O16^v^	0.79 (2)	1.85 (2)	2.6172 (16)	164 (2)
O6—H6*O*···O7^vii^	0.76 (2)	2.06 (2)	2.8035 (17)	166 (2)
O11—H11*O*···O22^viii^	0.80 (2)	2.39 (2)	3.0578 (16)	141 (2)
O11—H11*O*···O23^viii^	0.80 (2)	2.12 (2)	2.7906 (17)	142 (2)
O12—H12*O*···O9	0.81 (2)	2.05 (2)	2.8372 (15)	165.7 (19)
O13—H13*O*···O25^ix^	0.768 (18)	2.082 (19)	2.8027 (16)	156.5 (19)
O14—H14*O*···O4^vi^	0.77 (2)	2.18 (2)	2.9173 (16)	162 (2)
O15—H15*O*···O25^ix^	0.805 (19)	1.939 (19)	2.7296 (16)	167 (2)
O16—H16*O*···O32^ix^	0.74 (2)	2.00 (2)	2.7346 (18)	173 (2)
O21—H21*O*···O2	0.84 (2)	2.13 (2)	2.9010 (16)	153.9 (19)
O21—H21*O*···O3	0.84 (2)	2.28 (2)	2.8810 (17)	129.4 (18)
O22—H22*O*···O19^x^	0.765 (18)	1.995 (18)	2.7576 (15)	175 (2)
O23—H23*O*···O5^v^	0.83 (2)	2.01 (2)	2.8219 (15)	168 (2)
O24—H24*O*···O14^xi^	0.76 (2)	2.34 (2)	3.0445 (16)	156.3 (19)
O25—H25*O*···O5^v^	0.777 (19)	1.898 (19)	2.6709 (15)	173 (2)
O26—H26*O*···O31^v^	0.76 (2)	2.14 (2)	2.8463 (18)	155 (2)
O31—H31*A*···O18^xii^	0.700 (19)	2.08 (2)	2.7729 (17)	170 (2)
O31—H31*B*···O6^ix^	0.83 (2)	2.07 (2)	2.8493 (18)	156 (2)
O32—H32*A*···O26^ix^	0.89 (2)	1.89 (2)	2.7501 (17)	163 (2)
O32—H32*B*···O8^xii^	0.741 (18)	2.019 (18)	2.7471 (16)	167.1 (17)
O33—H33*A*···O27^iv^	0.833 (19)	2.16 (2)	2.9093 (18)	149.9 (19)
O33—H33*B*···O28^xii^	0.80 (3)	2.02 (3)	2.7371 (17)	150 (2)

Symmetry codes: (ii) *x*+1/2, −*y*+3/2, −*z*+1; (iv) *x*−1/2, −*y*+3/2, −*z*+1; (v) −*x*, *y*+1/2, −*z*+1/2; (vi) −*x*+1, *y*−1/2, −*z*+1/2; (vii) −*x*+1/2, −*y*+1, *z*−1/2; (viii) *x*, *y*−1, *z*; (ix) −*x*, *y*−1/2, −*z*+1/2; (x) *x*, *y*+1, *z*; (xi) −*x*+1, *y*+1/2, −*z*+1/2; (xii) *x*−1, *y*, *z*.
